# Enhancing the Efficacy of CAR T Cells in the Tumor Microenvironment of Pancreatic Cancer

**DOI:** 10.3390/cancers12061389

**Published:** 2020-05-28

**Authors:** Janina Henze, Frank Tacke, Olaf Hardt, Frauke Alves, Wa’el Al Rawashdeh

**Affiliations:** 1 University Medical Center Göttingen, Translational Molecular Imaging, Institute for Diagnostic and Interventional Radiology & Clinic for Hematology and Medical Oncology, 37075 Göttingen, Lower Saxony, Germany; janina.henze@stud.uni-goettingen.de (J.H.); falves@gwdg.de (F.A.); 2 Miltenyi Biotec B.V. & Co. KG, R&D Reagents, 51429 Bergisch Gladbach, North Rhine-Westphalia, Germany; Olaf@miltenyibiotec.de; 3 Charité University Medicine Berlin, Dept of Hepatology & Gastroenterology, 13353 Berlin, Germany; frank.tacke@charite.de; 4 Max Planck Institute for Experimental Medicine, Translational Molecular Imaging, 37075 Göttingen, Lower Saxony, Germany

**Keywords:** tumor microenvironment, pancreatic cancer, immunotherapy, CAR T cell therapy, extracellular matrix, cancer-associated fibroblasts

## Abstract

Pancreatic cancer has the worst prognosis and lowest survival rate among all types of cancers and thus, there exists a strong need for novel therapeutic strategies. Chimeric antigen receptor (CAR)-modified T cells present a new potential option after successful FDA-approval in hematologic malignancies, however, current CAR T cell clinical trials in pancreatic cancer failed to improve survival and were unable to demonstrate any significant response. The physical and environmental barriers created by the distinct tumor microenvironment (TME) as a result of the desmoplastic reaction in pancreatic cancer present major hurdles for CAR T cells as a viable therapeutic option in this tumor entity. Cancer cells and cancer-associated fibroblasts express extracellular matrix molecules, enzymes, and growth factors, which can attenuate CAR T cell infiltration and efficacy. Recent efforts demonstrate a niche shift where targeting the TME along CAR T cell therapy is believed or hoped to provide a substantial clinical added value to improve overall survival. This review summarizes therapeutic approaches targeting the TME and their effect on CAR T cells as well as their outcome in preclinical and clinical trials in pancreatic cancer.

## 1. Introduction

Pancreatic cancer, i.e., pancreatic ductal adenocarcinoma (PDAC), is a fatal disease with five-year overall survival rates of 1% to 5% and median survival duration of fewer than six months [1]. The poor prognosis has not substantially changed during the past decades, establishing pancreatic cancer as the fourth leading cause of cancer-related deaths in Western countries [2,3,4]. Therapeutic progress in other types of cancer will lead to its ascension in second place among all cancers within the next decade [5]. Surgery remains the only potentially curative treatment, but only a minority of patients show a resectable disease stage at diagnosis, due to invasion to the surrounding vasculature and due to lack of symptoms at an early stage [6]. Nonetheless, the median overall survival is still only 24 months for patients with resectable disease [7].

**Figure 1:**
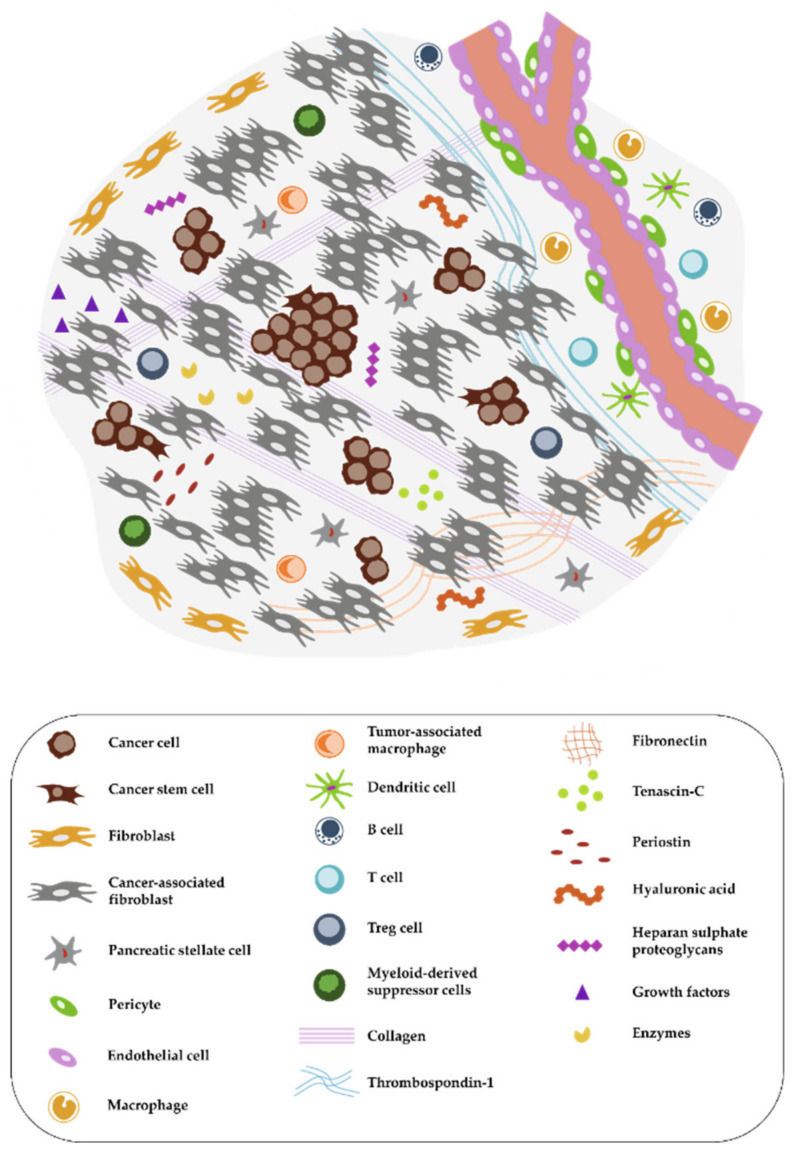
Complex tumor microenvironment (TME) of pancreatic cancer. The pancreatic ductal adenocarcinoma (PDAC) microenvironment is characterized by a dense desmoplastic stroma, with cancer-associated fibroblasts (CAFs) presenting the majority of the cell population (in grey). Tumor cells (round and brown) in aggressive PDACs can occur in tumor buds, small groups of cells, especially in the invasive front. A high abundance of extracellular matrix (ECM) molecules, enzymes, and growth factors is another important feature. Immune cells are often excluded from the TME or exhibit an immunosuppressive phenotype. The distribution of pro- and anti-inflammatory immune cells as well as the exact composition of the tumor stroma is dependent on the subtype of pancreatic cancer as discussed by Bailey et al. or by Karamitopoulou [12,15].

**Figure 2:**
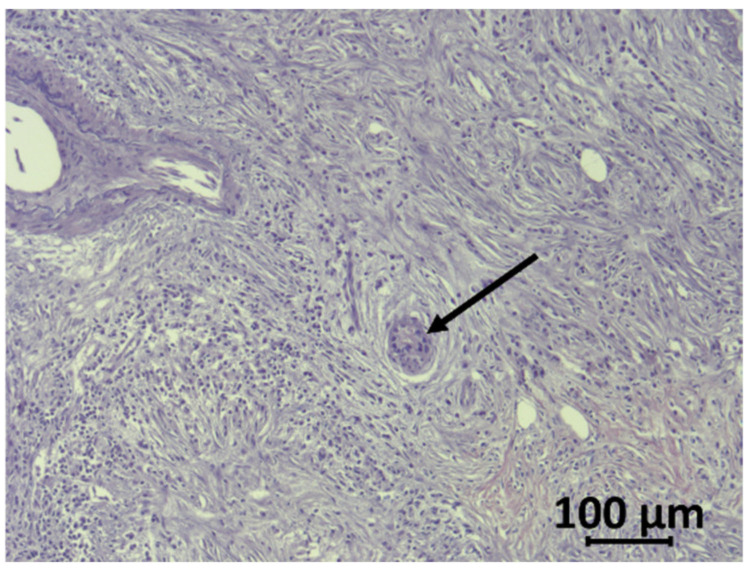
Haematoxylin/eosin-stained human PDAC sample. Tumor cells (arrow) are surrounded by the desmoplastic reaction of stromal cells and few immune cells.

Therapeutic failures of chemotherapy, targeted therapy, and immunotherapy of PDAC can be largely attributed to the special features of this cancer, which exhibits highly nutrient-poor, immunosuppressive, hypoxic and desmoplastic characteristics leading to rapid cancer progression [8]. The tumor is composed of only a minor number of malignant cells within a microenvironment of dense extracellular matrix (ECM), a barrier that prevents adequate drug delivery and might serve as a prognostic factor (Figure 1 and Figure 2) [8]. Responsible for the stromal reaction are mainly cancer-associated fibroblasts (CAFs) that develop from bone marrow-derived mesenchymal stem cells (MSCs), pancreatic stellate cells (PSCs), and quiescent resident fibroblasts through multiple pathways of activation [9]. The complex tumor vasculature in PDAC is characterized by a lack of blood vessels, leading to high levels of hypoxia in the tumor interior [10]. Furthermore, the capillaries and lymphatic vessels that are present tend to be collapsed due to high interstitial pressure, either from excess fluid or from solid stress [11]. Other non-neoplastic cancer-associated cells consist of immune-suppressor cells such as regulatory T cells (Treg), myeloid-derived suppressor cells (MDSC), and tumor-associated macrophages (TAM) that can inhibit CD8+ T cells, which play a key role in the antitumor immune response, and thereby establish an immunosuppressive tumor microenvironment [12]. Neural remodeling and perineural invasion (PNI), the neoplastic invasion of tumor cells into nerves, are further unfavourable histological features, and are considered as one of the main routes for cancer recurrence and metastasis after surgery [13]. Conventional therapies such as chemotherapy and radiation have focused on effective therapy of the malignant cell population. Thus, a concordant combination of various treatments targeting additional key cellular features of PDAC such as stroma, reversing suppressive immune reactions and enhancing antitumor reactivity may lead to more successful treatment strategies [14]. Thus, there is a clinically unmet need for new therapeutic options.

**Figure 3:**
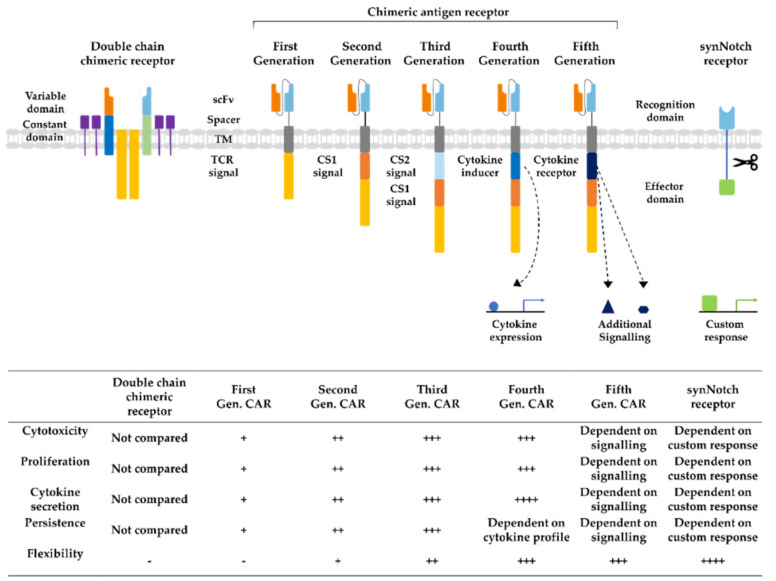
Developmental stages of chimeric antigen receptors. The first double chain chimeric receptors were engineered to customize the variable T cell receptor (TCR) domain by using V_H_ and V_L_ chains of antibodies (orange and bright blue boxes) fused to the constant regions of the TCR α- and β-chains (green and blue boxes). They mimicked the TCR in appearance and functionality. Activation relies on association with intracellular CD3ζ (yellow boxes), CD3γ, CD3δ, and CD3ε chains (purple boxes). The first generation of CARs combined the antigen recognizing scFv directly with the CD3ζ-signalling domain in one construct overcoming expression difficulties by the tremendous construct length of double chain chimeric receptors. Cytotoxicity, proliferation, cytokine secretion, and persistence of CARs were increased in second and third generation CARs by the addition of further costimulatory domains (CS1 and CS2) such as CD27, CD28, CD134, or 4-1BB. Introduction of T cell redirected for universal cytokine-mediated killing (TRUCKs) or fourth generation CARs increased the flexibility in CAR design for specific challenges even further, enabling local expression of cytokines such as IL-12, which are toxic in high concentrations. Fifth generation CARs, as fourth generation CARs, are based on second generation CARs. The individual antigen response is complemented by activation of intracellular domains of cytokines (dark blue box) e.g., IL-2Rβ, which induced signal transduction in the STAT3/5 pathway. Another group of artificial antigen receptors, gaining increased interest in recent years, are synNotch receptors. These receptors use the cleavage process after Delta-Notch binding and enable an unlimited variety of responses (green box) after target recognition such as cell fate determination with transcription factors and expression of selected cytokines or therapeutic antibodies. In this way, they bring the potential of immune cells as “living drugs” a big step forward.

Immunotherapy is a rapidly developing field within oncological research, especially since the development of chimeric antigen receptor (CAR) T cells, which are genetically engineered to express receptors targeting cancer cells for immunotherapy. CAR technology has made leaps of development since its conception in 1993, combining antigen recognizing regions from antibodies with intracellular T cell signaling domains (Figure 3) [16]. In this way, potential demasking of tumor cells by major histocompatibility complex (MHC) class I downregulation, can be overcome [17]. At first, double chimeric receptors were developed by engineering the V_H_ and V_L_ chains of immunoglobulins to the constant regions of the T cell receptor (TCR) [18]. Over time, CARs were modified into a single chain approach coupling a single chain variable fragment (scFv) derived from an antibody via a spacer and transmembrane domain to the CD3ζ signaling domain of the TCR [16]. The addition of costimulatory domains from CD28 or 4-1BB generated a stronger activating signal, circumventing the intracellular activation by TCR-domains, defining the second CAR generation [19]. Second-generation CARs targeting CD19 are the first CAR success-story wherein phase II study 81% of the B cell acute lymphoblastic leukemia patients demonstrated complete remission 28 days after infusion [20]. Their tremendous success in the treatment of leukemia and lymphoma patients led to the FDA approval of the first CAR T cell therapy as a second-line treatment in 2017 [21]. The incorporation of further costimulatory domains derived from CD27 or CD40 as well as the introduction of additional cytokine expression or induction of other signaling pathways established the third, fourth, and fifth generations of CAR T cells, increasing cytokine production, cell survival, and persistence [22]. In recent years, advanced CAR concepts, such as Tandem or Universal CAR approaches have been developed and enabled the targeting of challenging antigen expression profiles on cancer cells [23]. Other advanced CAR technologies explore mechanisms to switch on and off CAR expression on T cells to control possible toxic side effects [24]. Another upcoming class of engineered receptors is synthetic Notch (synNotch) receptors, which can induce transcriptional activation after target recognition [25]. Ultimately, all developmental generations of CARs offer various opportunities and challenges for prospective cell-based approaches as reviewed before [22,24,26]. 

Unfortunately, fewer exciting outcomes were achieved in initial clinical trials with CAR T cells targeting solid tumors, including PDAC. Successful CAR therapy for carcinomas needs to overcome the physical and environmental barriers in the tumor microenvironment (TME) [27]. The TME consists, next to tumor cells, of endothelial, immune, and inflammatory cells, stromal cells, the extracellular matrix and a broad spectrum of enzymes, cytokines, and growth factors [28]. This creates a strong physical barrier for CD8+ T cells, while their immune response is further diminished by the high amount of immunosuppressive immune cells present in the TME of PDAC [12,29]. These aspects must be considered and addressed in the field of cell-based immunotherapy against solid cancers. Here we review different strategies to overcome these hurdles for successful CAR T cell therapy in pancreatic cancer. 

## 2. CAR T Cells and the Tumor Microenvironment of Pancreatic Cancer 

### 2.1. CAR Targets for Pancreatic Cancer

The first obstacle for effective CAR T-cell therapy for carcinomas is the lack of suitable targets on carcinoma cells. CAR T therapy induces an ablation of all cells with a certain degree of antigen expression leading to potentially fatal side effects such as “on target/off tumor” toxicities [30]. Unfortunately, this is also the case for most of the PDAC targets tested in preclinical and clinical trials such as carcinoembryonic antigen (CEA), CD133, CD70, Claudin 18.2, epithelial cell adhesion molecule (EpCAM), receptor tyrosine-protein kinase erbB-2 (HER2), mesothelin, and prostate stem cell antigen (PSCA) (Table 1) [31].

The most advanced targets for clinical consideration are CEA and mesothelin, with up to five clinical trials completed, active, or recruiting (CEA: NCT03818165, NCT02850536, NCT02416466, NCT04037241, NCT03682744; mesothelin: NCT03323944, NCT03497819, NCT03638193, NCT01897415). In contrast, the only published results from clinical trials of CAR T cells in PDAC originate from mesothelin and CD133. The mesothelin-specific CAR trial resulted in two patients with a progression-free survival of four to five months and another patient showed a reduction of liver lesions, but not of the primary tumor (NCT01355965) [33]. The CD133 CAR trial also demonstrated a partial remission in two PDAC patients with Grade II toxicity, potentially due to the expression pattern of CD133 in hemopoietic stem cells (NCT02541370) [32]. Both studies verified the feasibility, safety, and principal efficacy of CAR T cell therapy for pancreatic cancer. Nevertheless, several problems prevented the induction of full remission and improvement of survival by immunotherapy despite its efficacy against metastases, often the discriminating factor for successful cancer therapy [71]. Two of the problems that must be solved for effective CAR T cell treatment are (i) emerging exhaustion and (ii) missing persistence of CAR T cells [32,33]. Co-treatment with PD-1/PD-L1 interfering checkpoint inhibitors or multiple infusions of CAR T cells might overcome these problems [72]. This aims to precondition chemotherapy and CAR constructs modifications, e.g., different costimulatory domains for CD4+ and CD8+ CAR T cells as well as transgenic cytokine expression, might overcome these problems [72]. However expression levels of cytokines need to be steered carefully, e.g., with conditional induction, to limit the risk for toxic cytokine release syndrome (CRS) [73].

The heterogeneity underlying PDAC makes therapeutic options based on one-size-fits-all approaches ineffective. Among others, Bailey et al. [15] defined for example four subtypes of PDAC, based on genomic analysis correlating with histopathological characteristics. These various PDAC types and their distinct stroma subtypes imply a specific stratification of the patients due to different behavior under the same treatment [74]. The complexity is further increased by another hurdle, which remains unchallenged: advanced targets in pancreatic cancer are usually heterogeneously expressed and are sometimes just present on 20% of the tumor cells, leading to progression of the diseases by the target-negative cells in the clinical trials [31,32,33]. Therefore, classifying patients in subtypes that could benefit from cell therapy would help improve outcomes and quality of life as well as avoid ineffective or even risky therapy approaches. These complex circumstances require the identification of new CAR targets as well as sophisticated Tandem, Universal CAR, and adapter-CAR approaches. In this way, unintentional “on target/off tumor” toxicities can be prevented for a safe and balanced application of CAR T cells in pancreatic cancer [75].

### 2.2. Targeting the Tumour Microenvironment in Pancreatic Cancer

A second major hindrance for cell therapy is the complex TME of solid tumors, representing an exceptional challenge in comparison to other tumor types. However, the histological key feature of PDAC is the occurrence of a unique desmoplastic reaction, comprising over two-thirds of the total tumor volume and destructing the architecture of normal pancreatic tissue [76]. Desmoplasia is marked by a dramatic increase in the proliferation of alpha-smooth muscle actin-positive fibroblasts and is also accompanied by the increased deposition of extracellular matrix molecules [77]. This has a strong impact on treatment outcomes since cytotoxic therapy can not only increase the amount of active CAFs but also increase their treatment resistance and tumor aggressiveness [78]. Another aspect of the dense tumor stroma is the limited availability of nutrients and oxygen [12]. The consequences of this deprivation for immune cells, including CAR T cells, in the stroma of solid tumors as well as major changes in the metabolic processes of the TME, have been extensively reviewed elsewhere [79,80,81] and will not be addressed in this review.

#### 2.2.1. Cancer-Associated Fibroblasts

Under normal conditions, stromal fibroblast cells communicate and interact with the surrounding ECM. They secrete and synthesize new ECM molecules as well as growth factors and enzymes, e.g., upon stimulation by tissue injury [82]. Under pathological conditions in the context of cancer however, the complexity of fibroblasts’ roles increases. In an early tumour stage, fibroblasts have been demonstrated to prevent tumour growth by remodeling the ECM and inducing an anti-tumour immune response [83]. Whereas at later stages with an established tumour, fibroblasts transform into activated CAFs, where they become tumorigenic and enhance metastasis-potential and chemoresistance [84]. ECM molecule expression and release of tumour-promoting cytokines can also be increased in activated CAFs, but stimuli and time point of phenotype switch are still under investigation [85]. CAFs can originate from various cell types, such as resident fibroblasts, chondrocytes, adipocytes, mesenchymal stem cells, pericytes, and mesenchymal transitioned endothelial and epithelial cells, including cancer cells and cancer stem cells [86]. In PDAC, CAFs can additionally be derived from PSCs, quiescent under normal conditions but transitioned into a myofibroblast-like phenotype under pathophysiological conditions in the pancreas [87]. Regardless of CAF origin, this cell type can constitute up to 90% of the tumour mass in PDAC, representing an inevitable hurdle for expedient treatment strategies [88].

Accordingly, numerous efforts have tried to dispose of CAFs or reprogram them within the TME [89]. In the context of CARs, several groups have generated fibroblast activation protein (FAP)-redirected CAR T cells to erase FAP-expressing CAFs, resulting in a reduction of ECM molecules and tumour growth, also in a syngeneic murine pancreatic cancer model [34,35,36]. FAP is a serine protease capable of local ECM modification by changing fibronectin orientation [90]. All studies emphasized the value of co-targeting CAFs and tumour cells simultaneously for solid tumours. Nevertheless, a debate is on-going regarding the safety of FAP as a CAR target, after the demonstration of hematopoietic side effects due to FAP+ bone marrow stromal cells (BMSCs) in mice [37,38]. Other possible extracellular markers expressed on CAFs, e.g., platelet-derived growth factor receptor (PDGFR) α and β, exhibit inappropriate expression patterns [86,91]. Therefore, more convenient and safe targets or target combinations need to be evaluated for successful CAF-redirected CAR establishment.

**Figure 4:**
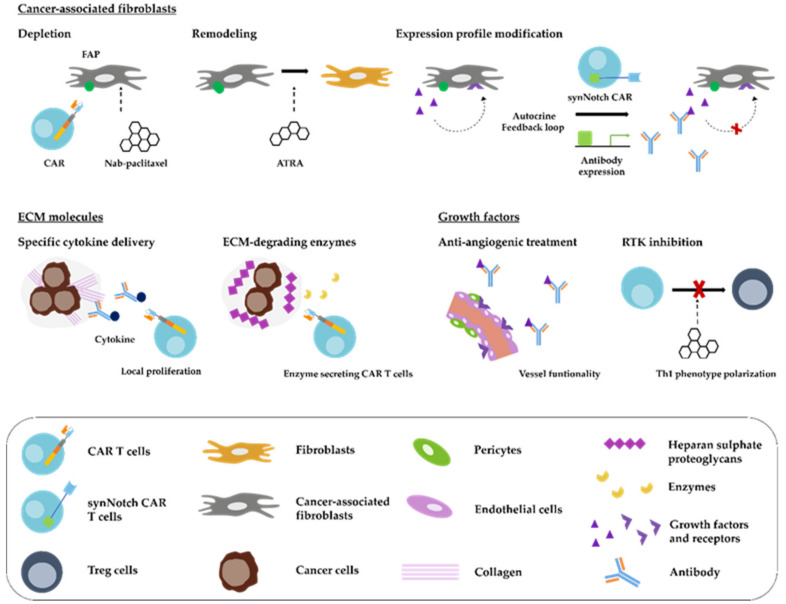
Strategies for CAR T cells to overcome or use the TME for successful immunotherapy. CAR T cells face major hinderances created by the distinctive TME of pancreatic cancer. Some of the hinderances might be surmounted or turned into a specific targeting strategy. CAFs may represent up to two thirds of the pancreatic tumor mass. However, CAF-depletion or remodeling approaches using CAR T cells or pharmacological substances such as ATRA or nab-paclitaxel might be able to break their crucial influence in the TME. Another strategy, potentially breaking the crucial influence of CAF expression profile in the TME, could be the application of FAP-redirected synNotch CAR T cells to deliver specific antibodies for inhibition of excess growth factors. Collagen is a key molecule in the creation of the dense ECM of PDAC, while its presence could be used for specific delivery of cytokines, required to boost CAR T cell efficacy and persistence. Moreover, it has already been demonstrated that CARs, re-equipped with ECM-degrading enzymes, such as heparanase, had higher infiltration compared to the control CARs. Multiple TME components have a high potential of influencing vessels development and growth. These components need to be targeted and modified, e.g., by inhibitory antibodies to improve vessel functionality and ensure directed CAR T cell transport to the pancreatic tumor. Use of broad RTK inhibition needs to be balanced after careful consideration of their influence on different TME players. In this way, polarization of pro-inflammatory cells into anti-inflammatory cells can be prevented.

Next to cell-based CAF depletion, drug-based therapeutic options have also been proposed. Nab-paclitaxel has been shown to decrease CAFs numbers in PDAC in a clinical trial in combination with gemcitabine (NCT00398086) [92]. Small molecules inhibiting the sonic hedgehog (SHH) pathway have demonstrated promising preclinical results but failed to recapitulate these outcomes in clinical trials [93,94]. A phase II clinical trial (NCT01130142, NCT01064622) with a combination of vismodegib (GDC-0449) and gemcitabine revealed no survival benefit [39]. One possible explanation supported by the results of Özdemir et al. [95] is that the depletion of myofibroblasts in pancreatic cancer may also accelerate cancer growth and reduce survival. While the myofibroblast-depleted tumours did not respond to gemcitabine, anti-CTLA4 immunotherapy inverted the outcome and resulted in prolonged animal survival. Although FAP+ cell-depletion upon adenoviral vaccination demonstrated an improvement of CD8+ T cell function [40], remodeling of CAF expression pattern instead of CAF depletion might be a better-suited strategy for combinatorial approaches with immunotherapy in PDAC. The clinically most advanced substance to alter CAF expression phenotype is all-trans retinoic acid (ATRA), currently used as the standard treatment of acute promyelocytic leukaemia but also tested in PDAC [96]. It reduces ECM and cytokine secretion by inhibiting FAP, ACTA2 and transforming growth factor β receptor (TGF-βR) expression on CAFs [41]. Suitability of ATRA for stromal remodeling in pancreatic cancer is currently under clinical investigation (NCT03307148, NCT03878524) [42]. Another preclinical substance reducing CAF activation and expression in PDAC is JQ1; an inhibitor of the BET family of bromodomain chromatin-modulating proteins [43]. JQ1 has been demonstrated to control MYC silencing [97]. Since MYC-activated cells secrete factors, which can induce an MYC-dependent metabolic program in CAFs, JQ1 might be able to interfere with the tumour cell-CAF crosstalk [44]. Furthermore, the PDAC-specific CAF precursor cells, PSCs, can be remodeled to decrease the desmoplastic reaction. Calpeptin, a calpain inhibitor, was also able to decrease fibrosis in a subcutaneous xenografts mouse model using co-implantations of PSCs and pancreatic cancer cells [45]. A combination of metformin and gemcitabine resulted in significantly lower tumour size and reduced collagen amounts in an orthotopic mouse model [98]. Unfortunately, most of the approaches are not protein or nucleic acid-based and cannot be produced by CAR effector cells. Therefore, FAP-depleting or remodeling molecules could be applied as a pharmacological pre-treatment to reshape the therapy-inhibiting expression pattern of CAFs. Alternatively, FAP-redirected CAR T cells could be used to deliver CAF remodeling factors or antibodies to inhibit the crucial expression profil of CAFs and their autocrine feedback loops (Figure 4) [99]. Tandem chimeric antigen or synNotch receptor approaches could be appllied simultaneously or in a time-shifted manner.

#### 2.2.2. Components of Extracellular Matrix in Pancreatic Cancer

One of the key features of activated fibroblasts is their distinct ECM production, especially crucial in PDAC with its pronounced desmoplasia [86]. Whatcott et al. [100] observed a strong negative correlation between patient survival and high levels of ECM deposition, also a solid tumour specific hurdle for immunotherapy [101]. Thus, the composition of the ECM in combination with the capability of CAR T cells to degrade extracellular matrix proteins can have a major influence on T cell tumour-trafficking and infiltration. A major challenge, however, is the fact that ECM proteins are not necessarily tumour-specific, but exert important physiological functions in organ development, tissue integrity, and wound healing [102].

Caruana et al. [46] demonstrated that ex vivo manipulated CAR T cells may downregulate ECM-degrading enzymes and overexpression heparanase improved CAR T cell infiltration and anti-tumour activity in vivo. However, heparan sulphate proteoglycans are not the only obstacle in the ECM of PDAC [103]. It is composed of collagens, non-collagen glycoproteins, glycosaminoglycans, growth factors, and proteoglycans as well as modulators of the cell-matrix interaction. Overexpressed ECM molecules, including thrombospondin, periostin, hyaluronic acid (HA), tenascin-C, vitronectin, collagens, and fibronectin increase pancreatic cancer cell migration and invasion [104]. Some of these molecules have already been exploited for possible effects on immunotherapy approaches. 

##### Structural protein

Collagen is the most frequent molecule in the ECM of PDAC and a major component of the desmoplastic reaction [105]. Furthermore, a collagen-derived proline can compensate as an alternative nutrient source in the resource-deprived TME [106]. However, collagen also regulates the activity, phenotype ratio and the amount of tumour-infiltrating T cells due to its dense network [107]. In this way, mammary tumours with a high collagen-density, correlated with a worse prognosis, contained a higher ratio of CD4+ to CD8+ T cells and an overall reduced amount of infiltrating CD8+ T cells. In PDAC, it was demonstrated that excessive collagen amounts abrogated tumour cell-directed movement of T cells by chemokines, but favoured T cell movement to the stroma cells in a contact guidance dependent manner [108]. These findings imply the relevance of the ECM composition for cell-based immunotherapy in solid tumours. Despite the severe impairment created by the collagen network, Ishihara et al. [47] managed to turn the presence of collagen into an advantage by increasing the delivery of cytokines with a short half-life, such as IL-2, and checkpoint inhibitors specifically and dosable to the TME through coupling to a collagen-binding domain. This enables a safe approach to shift the balance of pro- and anti-tumorigenic cytokines and stimulate the immune cells in the TME. Consequently, collagen-redirected IL-2 reduced common side effects such as vascular leak syndrome and increased tumour infiltrating CD8+ T cells in an orthotopic breast cancer mouse model [47].

##### Glycoproteins

Fibronectin, another common molecule in the ECM of pancreatic cancer, but not in healthy tissues, is considered to be a significant hallmark of epithelial-to-mesenchymal transition (EMT) occurring in advanced tumours [109]. Fibronectin interacts with many ECM and surface molecules, creating an active interaction platform. This stimulates the EMT and multiple aggressiveness- and resistance-related signalling pathways, which in turn upregulate fibronectin expression, resulting in a strong feedback loop in the TME [110]. As in the case of collagen, intratumoural regions with low fibronectin amounts displayed high leukocyte infiltration [111].The important role of fibronectin led to the creation of several approaches inhibiting its functions or using its presence in the TME for imaging, drug delivery, and therapy [112,113]. BC-1 coupled to IL-12 was used for TME-targeted cytokine delivery in clinical studies and resulted in stable disease in 46% of melanoma or renal cell carcinoma patients [48]. However, the single-chain variable fragment (scFv) L19-based cytokine delivery is more clinically advanced than the BC-1 based IL-2 delivery [49]. L19-IL2 (DARLEUKIN^®^) is already in clinical trials against various solid tumour types (NCT01058538, NCT02086721, NCT02735850, NCT03705403). Despite the promising preclinical results, a clinical trial of L19-IL2 with gemcitabine in patients with advanced pancreatic cancer had to be terminated due to lack of recruitment (NCT01198522). Nevertheless, phase II trials in melanoma patients resulted in reduced metastasis and increased survival demonstrating the potential of fibronectin-redirected IL-2 [50,51]. Besides IL-2, L19 was also coupled to IL-12 and tumour necrosis factor (TNF) α, revealing equally promising results in solid metastatic cancers [58,114], in particular for L19-TNF in combination with L19-IL2 [115]. In this way, targeting fibronectin enabled TME-specific cytokine delivery to outbalance immunosuppressive cytokines. This can be exploited as a combinatorial therapeutic strategy together with CAR T cells or as a pre-treatment.

Similar to fibronectin, tenascin-C is mostly present in the pathophysiological conditions of adults, building up a provisional matrix in the scar formation process [59]. It is upregulated in the ECM of solid tumours, including PDAC [60]. While the exact role of tenascin-C remains undefined, it is widely known for its modulation capacity on cell adhesion to fibronectin and its promotion of EMT, enhancing cancer cell growth and motility [116,117]. It has also been shown to interact with multiple ECM molecules and to facilitate the angiogenic switch by representing an important factor of the AngioMatrix (ECM and related protein involved in the angiogenic switch) inducing resistance to chemo- and anti-angiogenic therapy in PDAC [118]. Nevertheless, no correlation between high tenascin-C expression and survival has been determined. However, overexpression of tenascin-C together with other ECM-related factors has been shown to correlate with poor prognosis for patients of pancreatic cancer [119]. Tenascin-C pronounced importance in the context of solid tumours led to multiple approaches to modify tenascin-C in the ECM or to make use of its presence. Inhibition of tenascin-C expression is possible by blocking its natural activation pathways such as transforming growth factor β (TGF-β), but also by RNA interference resulting in only short survival prolongation [120]. Tenascin-C expression and signalling have been demonstrated to be prevented by angiotensin II type 1 receptor (AT-1) and angiotensin-converting enzyme (ACE) inhibitors, which has not yet been assessed in the clinic [120]. Another possibility would be to erase tenascin-C, as previously described for heparan sulphate proteoglycans, from the ECM of solid carcinomas, a process occurring after wound healing. Unfortunately, this mechanism has not yet been identified (reviewed by Spenle et al. [120]). Therefore, as in the case of fibronectin, multiple antibodies have been generated redirecting radionuclides and cytokines to the tenascin-C-rich ECM. F16-IL2 (TELEUKIN^®^), an IL-2 coupled antibody-cytokine fusion protein is the most advanced candidate with two clinical trials in solid tumours, such as breast and lung cancer (NCT01131364, NCT01134250). This recombinant protein demonstrated its ability to increase survival as well as the number of macrophages and NK cells in the tumour stroma in a BALB/c nude mice breast cancer model [52]. F16-IL2 clinical potency has also been analysed in a clinical setting in solid tumours including pancreatic tumours, demonstrating an anti-cancer activity in combination with doxorubicin [53]. 

Thrombospondin 1 (TSP-1) is a strong inhibitor of angiogenesis, promotes inflammatory (‘M1-type’) macrophage recruitment and prevents stemness of cancer cells. Via its crosslinking-interaction with the “don’t eat me”-signal CD47 it can directly induce tumour cell death [121,122]. However, it also releases the active form of TGF-β from its latent form, promotes Treg formation and inhibits T cell proliferation [123,124]. Several inhibitors for TSP-1 are available with the most advanced being ABT-510, CVX-045, and Trabectedin [62,63]. While ABT-510 showed a limited increase of cytotoxic T cell frequency, it did not demonstrate efficacy in various solid tumours as a monotherapy leading to its suspension from clinical development (NCT00586092) [62,125,126]. Trabectedin, approved for the treatment of sarcoma and ovarian cancer, indicated a tremendous effect on favourable cytokines/chemokine expression level, although there was no efficacy as a single agent in stage II clinical trial for salvage therapy in metastatic pancreatic cancer (NCT01339754) [64]. Nevertheless, based on the findings of Weng et al. [127] TSP-1-targeted therapy in combination with cell therapy may deserve a second chance as a more nuanced treatment. Here it was shown that downregulation of TSP-1 solely in dendritic cells increased the amount of tumour-infiltrating CD4+ and CD8+ T cells [127].

##### Glycosaminoglycan

Next to heparan sulphate proteoglycans, hyaluronic acid (HA) is another glycosaminoglycan, overexpressed in the ECM of PDAC [104]. HA is widely expressed in all tissues and plays an important role in multiple biological processes, e.g., cell proliferation, inflammation, and angiogenesis [128]. Nevertheless, it exerts its most important biological functions by regulating cell motility via CD44, the tissue hydration influencing the intestinal fluid pressure (IFP), tissue permeability, and drug delivery potential [100,129]. Consequently, high amounts of high molecular weight HA contribute to a stiff tumour matrix increasing the IFP and reducing the ability of chemo-, nanomedicine, and cell-based therapies to penetrate stroma-rich tumours [130]. Accordingly, HA accumulation in the ECM of pancreatic cancer patients correlates with poor survival [131]. Unlike tenascin-C, there is a specific way to remove excess high molecular weight HA from the ECM. HA disruption with the PEGylated human recombinant PH20 hyaluronidase (PEGPH20) indicated improved drug delivery and response in a mouse model of pancreatic cancer and increased CD8+ T cell infiltration and better checkpoint inhibitor efficacy in a syngeneic breast cancer mouse model [54,55]. PEGPH20 treatment also resulted in a remodeling of the TME by decreasing other ECM molecules, such as collagen and tenascin-C. The promising preclinical success was also transferred to the clinic (NCT03481920, NCT01453153, NCT01839487, NCT04058964, NCT03634332, NCT02241187, NCT02921022, NCT02910882, NCT01959139, NCT04134468, NCT03193190, NCT02715804) and was in stage III of clinical development for pancreatic cancer [56]. Unfortunately, the phase III study was not able to meet the endpoint criteria, halting further development [57]. Nevertheless, especially for cell-based therapy approaches, which are limited by larger diameters (hydrodynamic size) than chemotherapeutics, depletion of HA may have a potential of exerting a significant impact on therapy delivery. 

Altogether, these findings imply the importance of the ECM for the outcome of cancer therapy including immunotherapy. The impact of the ECM on the therapeutic outcome is further strengthened by the wide range of cytokines, which are bound and released by various ECM molecules after expression by CAFs and tumour cells, as recently reviewed by Tzanakakis et al. [132] for the group of the proteoglycans. Furthermore, options that failed before as monotherapies or in combination with chemotherapeutics deserve a second consideration for suitability in combination with immunotherapy. In the long-term, the latest CAR technologies could be utilized to secrete engineered proteins to increase tumoricidal immune response and CAR T cell infiltration, overcoming the complex barriers created by the ECM. 

##### Growth Factors in Pancreatic Cancer

The majority of the growth factors, expressed by cancer cells or CAFs in the TME, increase cell survival, proliferation, migration, and metastasis in an autocrine feedback loop or in a paracrine manner, via their associated receptors [99]. They can also be bound by ECM molecules and be released by enzymes, such as matrix metalloproteinases (MMPs) [86,133]. Aside from the close cancer cell and fibroblast communication network, some of these factors are also released by other immune cells in the TME, such as tumour-favouring M2 macrophages or neutrophils [134,135]. 

A thoroughly-investigated factor is the vascular endothelial growth factor A (VEGF-A), and its receptor (VEGFR2), which regulates the process of angiogenesis [28]. Unlike most hematologic malignancies, solid tumours heavily depend on the formation of new vessels for sufficient blood supply. Hypoxia in all tissues, including cells present in the intertumoral regions of PDAC, induces the expression of VEGF after hypoxia-inducible factor 1 alpha (HIF-1) translocation to the nucleus in a gradient manner, which in turn initiates the growth of new blood vessels into hypoxic regions [136,137]. Nevertheless, the relationship between angiogenesis and PDAC is far more complex. On the one hand, PSCs and CAFs secrete VEGF, which leads to increased, disorganized vascular growth and formation with enhanced IFP [11]. While on the other hand, the dense desmoplastic reaction around pancreatic tumours leads to vascular disruption, which further increases hypoxia and reduces drug administration [10]. This leads to insufficient therapeutic-dose delivery that might, to some extent, explain the low survival rates in patients with pancreatic cancer [61,138]. Cell therapy also relies on functioning vessels [79]. Fortunately, vessel function can be restored by using anti-angiogenic treatments, such as bevacizumab, to normalize vessel organization and IFP [139]. Co-treatment of angiogenesis inhibitor bevacizumab together with GD2-redirected CAR T cells increased tumour infiltration and antitumor activity in a preclinical neuroblastoma model [66]. Bevacizumab was already tested in pancreatic cancer patients in combination with gemcitabine. Despite the promising objective response rate of 21%, there was no difference in the overall survival time between the bevacizumab and the placebo group (NCT00088894) [67]. This undesirable outcome may be attributed to the ability of tumours to acquire resistance to VEGF inhibition, e.g., by the release of more proangiogenic factors, such as angiopoietin 1 (ANGPT1), resulting in increased amounts of vascular progenitor cells [140]. Recently, another mechanism dependent on the ECM molecule periostin, present in ECM of PDAC, has been revealed and induced revascularisation and macrophage recruitment [65]. The second effect was reversible by the addition of an anti-colony stimulating factor 1 receptor (CSFR1) antibody, blocking macrophage infiltration [65]. This highlights the importance of understanding the individual TME composition of each patient in order to match the most suitable anti-angiogenic treatment, because many of the early mentioned ECM molecules have been shown to modify angiogenesis in different ways, e.g., by VEGF interaction [129]. Modification of other TME molecules, such as thrombospondin-1, together with anti-angiogenic treatment has already been evaluated in the clinic by the co-treatment of advanced solid tumours with bevacizumab and ABT-510, resulting in partial response for one patient and stable disease for more than a year in five patients [68]. Hence, combining multiple anti-angiogenic approaches with cell therapy might be necessary for a successful cell-based immunotherapy of PDAC. These findings stress the importance of moving away from the current one-size-fits-all therapy approaches to more personalized combinatorial therapies, simulating personalized nanomedicine approaches [141].

Tumour cells in hypoxic areas often express other growth factors next to VEGF. Their interactions with their defined receptors lead to receptor tyrosine kinase (RTK) induction, which can be antagonized by the blockage of downstream signalling pathways with RTK inhibitors [142]. RTKs are a group of cell surface receptors involved in multiple key pathways of cell proliferation, differentiation, survival, and migration. The inhibition of the RTK, Axl, attracted attention for its influence on immune cells and not on tumour cells. Axl has been associated with the traditional RTK pathways in cancer cells and with the regulation of innate immune response and a more aggressive and resistant phenotype [143,144]. These findings motivated the preclinical evaluation of the Axl receptor as a target for monoclonal antibody immunotherapy in pancreatic cancer [145]. Small molecule inhibition by BGB324 of Axl decreased immune suppression and increased chemotherapy potency in pancreatic cancer and synergized with CAR T cell therapy in B cell malignancies [69,70]. This in vivo demonstrated synergy was dependent on T helper cell type 1 phenotype polarization, expressing an anti-tumorigenic cytokine profile, induced by Axl inhibition. Given the great influence on vessel functionality and further, on immune cells, growth factor modification might have a significant influence on the improvement of immunotherapy in solid tumours. These findings encourage the application of already clinically approved drugs as supporting combinatorial approaches with immunotherapy. Upon favourable outcomes from clinical trials, biological inhibitors such as bevacizumab, could even be secreted by the CAR T cells, creating a living drug.

## 3. Conclusions

Pancreatic cancer represents an exceptional challenge for successful cancer therapy. CAR T cells are no exception, instead, they face great obstacles but also have the capacity to offer valuable chances. Cell-based immunotherapy has shown pronounced clinical success in hematologic malignancies and its feasibility has been demonstrated in pancreatic cancer, but it needs to overcome certain barriers, such as infiltration, persistence, and exhaustion. However, the first major hurdle is the heterogeneity of pancreatic cancers in terms of proposed subtypes and varying target expression. This requires advanced CAR technology to ensure the successful targeting of all cancer cells. The complex and heterogenous TME is the second major hurdle specifically for CAR T cells against pancreatic cancer. All parts of the TME require individual strategies. Reprogramming of CAFs might be more favorable than CAFs depletion without directly powering up the therapy intensity. The presence of tumor specific ECM molecules, as described in this review, would enable a specific delivery of cytokines, using agents such as F19-IL-2 [53]. In this way, both approaches could be combined strategically to first loosen the dense stroma, before boosting up CAR T cells. This represents an option to increase the temperature of immunological “cold” tumors, similar to PDACs [146]. However, tremendous tumor growth in areas that are no longer suppressed needs to be vigorously prevented. The same holds true for situations, where CAR T cells are equipped with ECM-degrading enzymes, such as overexpressed heparanase, or tumors are pre-treated with IFP decreasing molecules such as PEGPH20 [55,56]. Restored baseline IFP and vessel function is of major importance for successful CAR T cell delivery to the tumor, even if they are provided with infiltration-increasing mechanisms, such as heparanase [46]. IFP and enhanced permeability and retention (EPR) effect in cancer nanomedicine are closely related. Hence, high-resolution 3D imaging techniques, used in nanotherapy, could be applied for translational approaches in terms of vessel functionality in vivo and later patient stratification for combinatorial cell-based therapies [147]. 

A high need for vessel functionality assessment is also present for the analysis of the interplay of all the ECM molecules and growth factors in the TME, which can influence vessel growth and development [11,68]. Tumors undergoing anti-angiogenic treatment strategies, such as bevacizumab, may develop resistance mechanisms. Those mechanisms can be dependent on the ECM composition, but might also be overcome by modifications of the present molecules. The availability of vessel-independent growth factors, secreted by the various players in the TME indicates a medical need for in-depth patient-stratifications based on the presence of key different TME molecules, especially when it comes to the application broad range RTK inhibitors. This research requires technically advanced organoid or tissue printing methods, combined with established immunological assays. 

**Figure 5:**
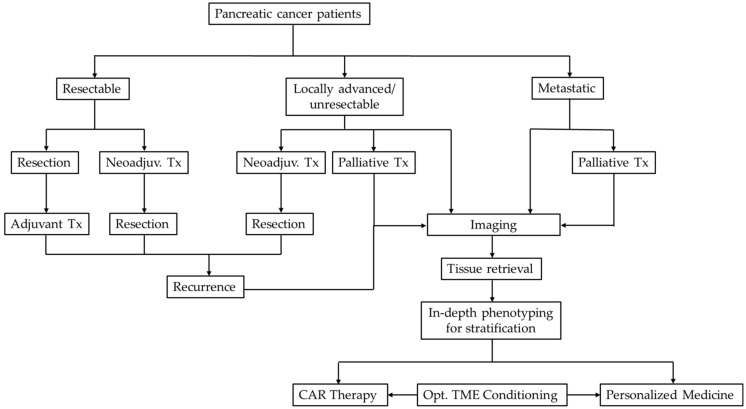
Strategy flow chart for PDAC therapy. Pancreatic cancer patients are classified into one of three categories upon diagnosis. Therapy (Tx) is chosen on the basis of this classification. In case of later stage PDAC or recurrent tumor, personalized medicine approaches could be of use. Imaging of patients would be followed by tissue retrieval to perform in-depth phenotyping of the tumor and its stroma. This could be performed by the application of up and coming technologies such as patient-derived organoids analysis, RNA-Seq or multiplex immunofluorescence staining. All in all, such refined selection criteria enables the balanced and careful stratification of patients into further effective and safe therapy paths, including personalized therapy approaches, such as CAR T cell therapy, with or without conditioning of the tumor microenvironment.

Taken together, there is an overall need for the development of new in vivo and in vitro assays in combination with imaging strategies to facilitate combinatorial research and improve preclinical translation potential. Agents, which might have failed as monotherapies, might deserve a second look in the context of combinatorial approaches with immunotherapy, due to their characteristics as a “living drug”. Research on different TME subtypes needs to be intensified and these parameters, in addition to molecular markers, need to be taken into account to define clear subgroups of PDAC. The acquired knowledge should assist in identifying only the PDAC patient, who will benefit from a particular personalized medicine concepts (Figure 5).  Therefore, sub classifying patients would help to improve outcomes and quality of life, as well as avoid ineffective therapy and reduce financial and organizational burdens on the health systems, healthcare providers, and the patients. These efforts will hopefully utilize existing and developing pharmacological therapies, regardless of their stand-alone therapeutic success, in combination with CAR T cells to create highly improved multifactorial therapeutic strategies, that can overcome the current hurdles faced by the challenging TME in pancreatic cancer. 

## Figures and Tables

**Table 1:** Therapeutic options for combinatorial stromal and immunotherapy.

Therapeutic	Proposed Effect	Clinical Trials	References
**2.1. CAR T Cell Therapy for Pancreatic Cancer**			
CEA	CAR Target	Pancreatic cancer: NCT03818165, NCT04037241, NCT02850536, NCT02349724, NCT03682744, NCT03267173, NCT02416466, NCT02959151	[31]
CD133	CAR Target	Pancreatic cancer: NCT02541370	[31,32]
CD70	CAR Target	Pancreatic cancer: NCT02830724	[31]
Claudin 18.2	CAR Target	Pancreatic cancer: NCT03890198, NCT03302403	[31]
EpCAM	CAR Target	Pancreatic cancer: NCT03013712	[31]
HER-2	CAR Target	Pancreatic cancer: NCT02713984, NCT03267173	[31]
Mesothelin	CAR Target	Pancreatic cancer: NCT02706782, NCT03267173, NCT03497819, NCT03638193, NCT01897415, NCT01583686, NCT02465983, NCT03323944, NCT02959151, NCT02580747	[31,33]
PSCA	CAR Target	Pancreatic cancer: NCT03267173, NCT02744287	[31]
**2.2.1. Cancer-Associated Fibroblasts**			
FAP-CAR T cells	CAF depletion	Solid tumors: NCT03932565, NCT01722149, NCT03050268	[34,35,36,37,38]
Vismodegib	CAF depletion	Pancreatic cancer: NCT01195415, NCT01064622, NCT01537107, NCT01088815, NCT00878163, NCT01713218, NCT02465060	[39]
CAF vaccine	CAF depletion	N/A	[40]
ATRA	CAF remodeling	Pancreatic cancer: NCT03307148, NCT03878524	[41,42]
JQ1	CAF remodeling	N/A	[43,44]
Calpeptin	CAF remodeling	N/A	[45]
**2.2.2. Components of Extracellular Matrix in Pancreatic Cancer**			
Heparanase-expressing CAR T cells	Heparan sulphate proteoglycans degradation	N/A	[46]
CBD-IL-2/CBD-CPI	Collagen redirected delivery	N/A	[47]
BC-1	Fibronectin redirected delivery	N/A	[48]
DARLEUKIN	Fibronectin redirected delivery	Pancreatic cancer: NCT01198522 Solid tumors: NCT01058538, NCT02086721, NCT02735850, NCT03705403	[49,50,51]
TELEUKIN	Tenascin-C redirected delivery	Solid tumors: NCT01131364, NCT01134250	[52,53]
PEGPH20	Hyaluronic acid degradation	Pancreatic cancer: NCT03481920, NCT01453153, NCT01839487, NCT04058964, NCT03634332, NCT02241187, NCT02921022, NCT02910882, NCT01959139, NCT04134468, NCT03193190, NCT02715804	[54,55,56,57]
ABT-510	Thrombospondin 1 inhibition	Pancreatic cancer: NCT00586092 Solid tumors: NCT00113334, NCT00073125, NCT00061646	[58,59,60,61]
CVX-045	Thrombospondin 1 inhibition	Solid tumors: NCT00879554	[62,63]
Trabectedin	Thrombospondin 1 inhibition	Pancreatic cancer: NCT01339754 Solid tumors: NCT00002904, NCT00786838, NCT03127215, NCT01273480, NCT01267084	[62,63,64]
MZ-1	Periostin inhibition	N/A	[65]
**2.2.3. Growth Factors in Pancreatic Cancer**			
Bevacizumab	VEGF) inhibition	Pancreatic cancer: NCT00614653, NCT00365144, NCT00088894., NCT00112528, NCT00366457, etc.	[66,67,68]
BGB324	Axl RTK inhibition	Pancreatic cancer: NCT03649321	[69]
TP-0903	Axl RTK inhibition	N/A	[70]

Abbreviations: CEA, carcinoembryonic antigen; EpCAM, epithelial cell adhesion molecule; HER-2, receptor tyrosine-protein kinase erbB-2; PSCA, prostate stem cell antigen; FAP, fibroblasts activation protein; CAR, chimeric antigen receptor; CAF, cancer-associated fibroblasts; ATRA, all-trans retinoic acid; N/A, not applicable; CBD, collagen binding domain; CPI, immune checkpoint inhibitors; PEGPH20, PEGylated recombinant human hyaluronidase; VEGF, vascular endothelial growth factor; RTK, receptor tyrosine kinase.
